# 广州女性低剂量CT肺癌筛查结果 及高危因素探索

**DOI:** 10.3779/j.issn.1009-3419.2024.101.14

**Published:** 2024-05-20

**Authors:** LU Xuanzhuang, QIU Qiuxia, YANG Chunyu, LI Caichen, LI Jianfu, XIONG Shan, CHENG Bo, ZHOU Chujing, DU Xiaoqin, ZHANG Yi, HE Jianxing, LIANG Wenhua, ZHONG Nanshan

**Affiliations:** ^1^510120 广州，广州医科大学附属第一医院（卢炫庄，邱秋霞，杨春玉，黎才琛，李坚福，熊珊，程博，周楚静，杜小芹，何建行，梁文华，钟南山）; ^1^The First Affiliated Hospital of Guangzhou Medical University, Guangzhou 510120, China; ^2^510120 广州，广州呼吸健康研究院（卢炫庄，邱秋霞，杨春玉，黎才琛，李坚福，熊珊，程博，周楚静，杜小芹，何建行，梁文华，钟南山）; ^2^Guangzhou Institute of Respiratory Health, Guangzhou 510120, China; ^3^511436 广州，广州医科大学南山学院（卢炫庄）; ^3^Nanshan School, Guangzhou Medical University, Guangzhou 511436, China; ^4^510080 广州，广州市卫生健康委员会（张屹）; ^4^Guangzhou Municipal Health Commission, Guangzhou 510080, China; ^5^510515 广州，南方医科大学（何建行）; ^5^Southern Medical University, Guangzhou 510515, China

**Keywords:** 肺肿瘤, 低剂量CT, 早期筛查, 高危因素, Lung neoplasms, Low-dose CT, Early detection, Risk factors

## Abstract

**背景与目的:**

肺癌高居中国恶性肿瘤发病率和死亡率之首。既往肺癌筛查试验大多为针对吸烟者等高危人群的选择性筛查，然而亚洲肺癌病例非吸烟女性占相当比例。本研究旨在评估广州市社区女性的肺癌患病率并探究肺癌高危因素。

**方法:**

采用胸部低剂量计算机断层扫描（low-dose computed tomography, LDCT）对广州市40-74岁女性居民行肺癌筛查，并根据2018版《中国肺癌低剂量螺旋CT筛查指南》对肺结节进行分类管理及计算阳性结节和肺癌检出率。筛查对象在行检查前均须完成“肺癌危险因素调研问卷”，后对问卷危险因素进行最小绝对收缩和选择算子惩罚Logistic回归分析。

**结果:**

分析共纳入6256名女性，发现阳性结节1228例（19.63%），确诊肺癌117例（1.87%），其中原位癌6例，I期103例（占肺癌比例88.03%）。根据问卷变量行最小绝对收缩和选择算子（Least absolute shrinkage and selection operator, LASSO）惩罚Logistic回归分析发现，年龄≥50岁（OR=1.07, 95%CI: 1.06-1.07）、个人恶性肿瘤史（OR=3.29, 95%CI: 3.22-3.37）、纺织职业（OR=1.10, 95%CI: 1.08-1.13）、小时候使用煤为燃料（OR=1.14, 95%CI: 1.13-1.16）以及食物过敏史（OR=1.10, 95%CI: 1.07-1.13）是该地区女性肺癌的高危因素。

**结论:**

胸部LDCT可以发现大量早期肺癌，可用于女性肺癌筛查。年龄≥50岁、个人恶性肿瘤史、纺织职业、小时候使用煤为燃料和食物过敏史是该地区女性人群肺癌的高危因素，需提高该群体对肺癌早筛的重视程度。

## Abstract (en)

**Background and objective:**

Both of lung cancer incidence and mortality rank first among all cancers in China. Previous lung cancer screening trials were mostly selective screening for high-risk groups such as smokers. Non-smoking women accounted for a considerable proportion of lung cancer cases in Asia. This study aimed to evaluate the outcome of community-based mass screening in Guangzhou and identify the high-risk factors for lung cancer.

**Methods:**

Residents aged 40-74 years in Guangzhou were screened with low-dose computed tomography (LDCT) for lung cancer and the pulmonary nodules were classified and managed according to China National Lung Cancer Screening Guideline with Low-dose Computed Tomography (2018 version). The detection rate of positive nodules was calculated. Before the LDCT examination, residents were required to complete a "lung cancer risk factors questionnaire". The risk factors of the questionnaire were analyzed by least absolute shrinkage and selection operator (LASSO) penalized Logistic regression analysis.

**Results:**

A total of 6256 residents were included in this study. 1228 positive nodules (19.63%) and 117 lung cancers were confirmed, including 6 cases of Tis, 103 cases of stage I (accounting for 88.03% of lung cancer). The results of LASSO penalized Logistic regression analysis indicated that age ≥50 yr (OR=1.07, 95%CI: 1.06-1.07), history of cancer (OR=3.29, 95%CI: 3.22-3.37), textile industry (OR=1.10, 95%CI: 1.08-1.13), use coal for cooking in childhood (OR=1.14, 95%CI: 1.13-1.16) and food allergy (OR=1.10, 95%CI: 1.07-1.13) were risk factors of lung cancer for female in this district.

**Conclusion:**

This study highlighted that numerous early stages of lung cancer cases were detected by LDCT, which could be applied to screening of lung cancer in women. Besides, age ≥50 yr, personal history of cancer, textile industry and use coal for cooking in childhood are risk factors for women in this district, which suggested that it’s high time to raise the awareness of early lung cancer screening in this group.

目前，癌症依然是全世界的主要死因之一。2022年，癌症导致全球近六分之一（近1000万）人口死亡，肺癌的死亡人数位居癌症死亡谱首位，并且新发肺癌病例仍逐年上升^[1,2]^，为家庭及国家卫生经济造成沉重负担^[3]^，是我国人民生命健康的巨大威胁。2015年广州市肺癌发病率达58/10万，死亡率达47/10万^[4,5]^，严重危害广州市居民的健康。

由于肺癌的起病较为隐匿，早期没有典型症状，临床确诊时以晚期居多，我国肺癌年龄标化的5年生存率在2018-2020年（山东地区）仅为24.4%^[6]^，预后极差。而0-I期肿瘤患者在完全切除肿瘤后的5年生存率可达到或接近100%^[7]^，预后明显改善。胸部低剂量计算机断层扫描（low-dose computed tomography, LDCT）对于肺癌筛查有较高的灵敏性，相较胸片能显著增加肺癌（尤其是I期肺癌）的检出率，2021年《中国肺癌筛查与早诊早治指南》与全球肺癌筛查指南及专家共识一致表明LDCT为目前最佳筛查手段^[8⇓⇓⇓⇓⇓⇓⇓⇓-17]^。采用LDCT进行肺癌筛查并对疑似恶性病灶进行早期干预，能有效降低肺癌相关死亡率，改善生存状况^[18]^。因此，通过肺癌筛查实现“早诊早治”是改善肺癌的预后、提高生存率、降低死亡率的重要措施^[19]^。

以往的肺癌筛查项目主要针对高危人群，特别是吸烟者^[19⇓⇓-22]^，而不吸烟的年轻人通常被排除在现有筛查指南之外^[23⇓-25]^。然而，近年来的研究^[25⇓-27]^开始关注年轻女性和不吸烟人群中肺癌的增加，尤其是发现非吸烟女性中肺癌病例逐渐增多，其病因尚未完全明了。研究^[28,29]^显示，在东亚和南亚，60%-80%的非小细胞肺癌（non-small cell lung cancer, NSCLC）女性患者从未吸烟。尽管如此，我国目前尚无专门针对女性群体的肺癌筛查研究。目前研究^[30,31]^表明，燃煤和烹饪油烟可能是不吸烟女性中肺癌发病的关键因素，但对于女性肺癌的更多高危因素还需进一步探索。因此，分析女性筛查人员的肺癌发病率以及探究女性肺癌的高危因素对于制定符合我国乃至亚洲非吸烟女性的肺癌筛查标准具有重要意义。

本研究基于广州市越秀区“爱肺计划”，该项目使用LDCT对广州市越秀区4条街道居民进行肺癌筛查，本研究拟探讨该地区40岁以上女性人群的肺癌发病率及高危因素。

## 1 资料与方法

### 1.1 研究对象 “

爱肺计划”项目采用LDCT进行肺癌筛查，于2015年12月至2021年7月完成受试者的招募和筛选，并对其跟踪调查至2022年8月，以获得肺癌确诊信息。为了招募研究中的参与者，项目团队采用多种方法，包括分发信息传单、通过电话和电视广播进行询问、定期组织以肺癌筛查为重点的健康论坛、通过社交媒体平台进行推广、举办一系列教育研讨会和网络研讨会、定制宣传材料以满足不同人群的需求等，并与社区组织建立合作伙伴关系，以促进宣传和转诊。有兴趣参与的个人在社区中心登记个人详细信息，并填写知情同意书。4个街道的筛查在5年半时间里广泛宣传和通知，对自愿入组的志愿者进行一次CT检查。4位女性居民多次参与筛查，分析数据时已剔除，确保入组人员在5年半期间无重复。（1）入选标准：①广州市越秀区人民街、北京街、珠光街、光塔街共4条街道40-74岁女性常住居民，年龄参考既往研究和指南推荐^[17,32⇓-34]^，常住居民定义为连续居住半年（包括半年）以上；②同意接受LDCT扫描。（2）排除标准^[19,35]^：①过去5年内被诊断为癌症或接受癌症相关治疗（非黑色素瘤皮肤癌除外）；②过去1年内做过胸部CT；③有癌症相关症状（如咯血、呼吸困难、无法爬两层楼梯）。各环节脱落人数见图1。本研究经广州医科大学第一附属医院医学伦理委员会批准通过（批号：YKLS2015-25），所有参与者均已自愿签署知情同意书。

**图 1:**
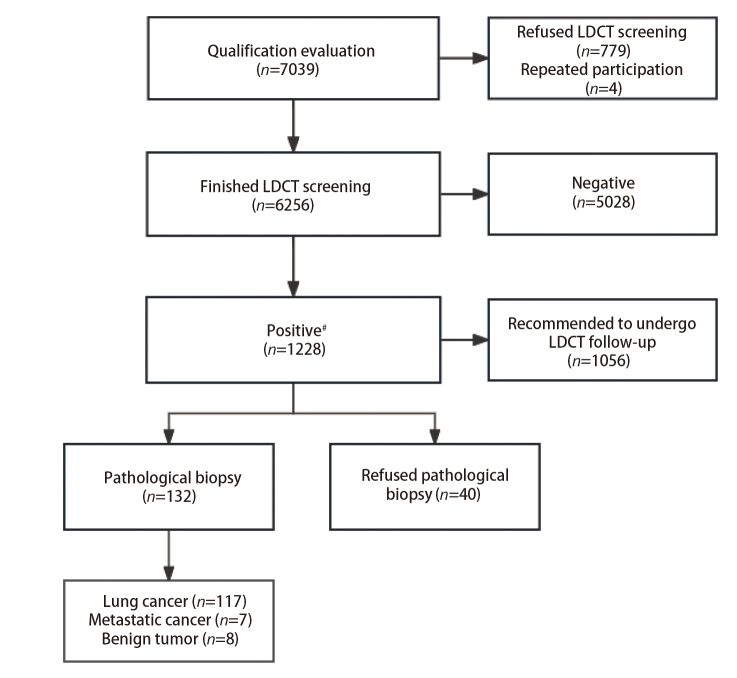
参与LDCT筛查流程图（不包括间隔癌）

### 1.2 研究方法

#### 1.2.1 问卷调查

在完成知情同意后，所有参与者均需完成一份自我报告问卷（具体问卷及变量的客观定义见 http://www.lungca.org/files/2024s23survey.doc），其中包括人口统计学、吸烟史、职业暴露、个人和家庭病史的详细信息。该试验已在ClinicalTrails.gov注册（No.NCT04938804），并根据方案进行。

#### 1.2.2 筛查方法和阳性判定标准

完成问卷后1个月内，所有参与者前往广州医科大学第一附属医院进行LDCT扫描。仪器采用德国埃尔朗根西门子Definition AS+ 128或256排螺旋CT扫描仪，所有受检者均取仰卧位，双手上举，于吸气末单次屏气完成扫描，扫描范围为肺尖至后肋膈角尖端水平。设置管电压100-110 kV，管电流30 mAs，薄层重建层厚为1.0 mm。根据2018版《中国肺癌低剂量螺旋CT筛查指南》的建议^[36]^，筛查阳性判定标准为直径≥8 mm纯磨玻璃结节或直径≥5 mm部分实性和实性非钙化结节以及≥30 mm肿块。阳性结节检出率计算公式为：阳性结节检出率（%）=（检出阳性结节人数/总筛查人数）×100%。

#### 1.2.3 诊治与随访

我们对不确定病变的受试者进行连续LDCT随访，阳性筛查结果的管理由经验丰富的胸科临床医生在Fleischner学会和中华医学会共识的指导下进行^[34,37]^，胸科临床医生将会慎重考虑对这些受试者进行包括高分辨CT、正电子发射型计算机断层显像（positron emission computed tomography, PET）/CT、有创性检查等诊断检查。所有患者术前均予进行多学科会诊。病理诊断将作为金标准进行组织学分型及分期，可分为腺癌（包括管状腺癌、乳头状腺癌、细支气管癌、肺泡细胞癌等）、鳞癌（包括梭形细胞癌、淋巴上皮癌、基底细胞癌等）、大细胞肺癌和小细胞肺癌。分期的依据是美国癌症联合委员会（American Joint Committee on Carcer, AJCC）和国际癌症控制联盟（Union for International Cancer Control, UICC）制定的第8版肺癌肿瘤原发灶-淋巴结-转移（tumor-node-metastasis, TNM）分期系统^[38]^。活检结果由2名病理学专家解释，他们对患者的临床特征和血清测量结果不知情。随后临床医生将综合我国的肺癌临床诊疗指南^[39]^及国际肺癌研究的进展^[40]^对肺癌确诊患者的治疗方案进行临床决策。各年龄段肺癌检出率计算公式为：各年龄段肺癌检出率（%）=（该年龄段确诊人数/该年龄段总筛查人数）×100%。

项目工作人员对阴性结果的参与者也进行了跟踪随访，记录筛查后的管理和生存情况，跟踪方式包括但不限于电话随访、社区走访等。数据分析截止日期为2022年8月31日，中位随访时间为3.4年（IQR：2.7-4.5年）。失访率在前12个月为0.7%，截止24个月时增加到1.6%，到截止日期累计达7.1%。失访的主要原因是对辐射暴露的担忧、后续随访的经济负担大、对随访电话的不响应以及没有明确原因的自愿退出。值得注意的是，间隔癌定义为行基线LDCT筛查时未出现（或未被发现）阳性结节但2年内病理确诊为癌^[35,41]^。疑似病例定义为存在阳性结节并被临床医生推荐进行病理活检但拒绝接受活检的人群。

#### 1.2.4 质量控制

（1）广州医科大学第一附属医院对所有工作人员进行资质审核并开展针对性的技术培训，包括临床筛查技术培训，提高工作人员（包括调查人员、技术人员）对肺癌早筛的认识和职业能力，确保参与者获得正确的问卷填写指引以及后续随访质量，避免漏诊误诊。（2）定期进行质控，严格控制数据质量，完成问卷后须经质控员确认方可整理归档，且最终剔除回答数<20%的变量。在本研究中，只有2个变量（初婚年龄和哮喘患者在地面行走或爬楼梯时呼吸短促）由于缺失值超过20%而最终被排除，其中初婚年龄缺失比例为1.66%（1448/6256），哮喘患者在地面行走或爬楼梯时呼吸短促缺失比例为37.23%（51/137），阳性病例和确诊病例有个别筛查者缺失问卷填写，但比例不满10%，因此用MICE包多重插补法补充完整^[42,43]^。（3）LDCT筛查结果由经验丰富的影像医学专科医师进行阅片，若无法明确判定，则交由高年资的副主任或以上职称影像医学专科医师进行复核。

#### 1.2.5 统计学方法

肺癌确诊数据的截止时间为2022年8月。对于正态分布与非正态分布的连续变量分别采用均数（标准差）及中位数（四分位间距）表示，并使用t检验或秩和检验比较组间差异。分类变量采用例（百分数）表示，并使用Pearson’s卡方检验比较组间差异。未进行多重假设检验校正。我们以是否确诊肺癌（含/不含疑似病例）作为因变量，调查问卷纳入的危险因素作为自变量，对问卷危险因素进行单因素Logistic回归分析初步探究女性肺癌的高危因素。鉴于经最小绝对收缩和选择算子（least absolute shrinkage and selection operator, LASSO）筛选危险因素后使用Logistic回归分析时，可能在患病率低时出现错误的系数估计，LASSO惩罚Logistic回归可以有效避免错误的系数估计以及多重共线性问题^[44,45]^。因此，本研究使用LASSO惩罚Logistic回归建立了肺癌风险（不包括间隔癌）的预测模型，探究与女性肺癌有关的高危因素，并以优势比（odds ratio, OR）及95%置信区间（confidence interval, CI）为效应评估指标。LASSO是一种缩小所有回归系数的惩罚方法，将许多不相关的特征系数设置为零，以避免过拟合并保留有价值的变量。我们在预测模型中纳入了由自我报告问卷确定的变量。为了确保模型的稳健性，我们将70%的参与者随机分配到训练集，其余30%分配到内部验证集。我们在训练集上使用最小标准进行10次交叉验证，以确定惩罚参数λ的最优值。我们将训练过程中得到的惩罚Logistic模型应用于验证样本，以计算肺癌的预测概率。模型判别通过曲线下面积（area under the curve, AUC）进行评价。最终模型包括预测变量，可以表示为具有以下结构的线性函数：线性预测器=截距+系数1*变量1+系数2*变量2+系数3*变量3+…+系数x*变量x。为了确保准确性，我们排除了所有缺失值超过20%的变量。多重插补法仅用于缺失数据小于20%的变量。本研究采用SPSS 26.0统计学软件及R（版本：4.1.2）运行R包“glmnet”（版本：4.3.1）进行数据分析，并用R包“ggplot2”（版本：3.4.0）进行绘图。所有统计学检验均为双侧检验，P<0.05为差异具有统计学意义。

## 2 结果

### 2.1 参与筛查人员基线信息和筛查结果

最终完成肺癌筛查的女性居民共计6256人。平均年龄（57.5±8.8）岁，其中40-49岁、50-59岁和≥60岁年龄组女性的人数（占比）分别为1362人（21.77%）、2037人（32.56%）和2857人（45.67%）。共发现阳性结节1228例，阳性检出率为19.63%，其中确诊肺癌117例（不含间隔癌）（9.53%）。在确诊肺癌病例中，Tis期6例（5.13%），I期103例（88.03%）[含T1a(mi)期34例，占I期比例33.01%]，II期1例（0.85%），III期3例（2.56%），IV期4例（3.42%）。总体而言，肺癌的确诊率随着年龄的增长呈逐步增加趋势，40-49岁为0.81%，50-59岁为1.52%，≥60岁为2.62%。其中，除了IB期在40-49岁年龄段确诊率最高（0.15%），Tis、T1a(mi)、IA、IIB、IIIB和IV期均在≥60岁年龄段的确诊率最高，分别为0.18%、0.63%、1.47%、0.04%、0.04%和0.14%。各年龄段T1a(mi)和IA期肺癌检出率均远高于其他分期（除40-49岁年龄段IA与IB期相同）（见表1和图2）。在确诊的117例肺癌中，除了4例（3.42%）病理类型未知，其余113例（96.58%）均为肺腺癌。其中早期和局部晚期肺癌（Tis-IIIA期，共112例）均以手术治疗切除结节病灶，并基于术后病理结果根据主诊医生建议完成后续辅助治疗。病理IIIB期的1例患者采用新辅助免疫治疗联合手术治疗，术后予免疫辅助治疗维持。病理IV期的4例患者为全身抗肿瘤治疗。值得注意的是，发现间隔癌2例，I期1例，IV期1例。

**表 1:** 各年龄段女性肺癌筛查结果[n (%)]

Stage and histologic type	Total (n=6256)	40-49 yr (n=1362)	50-59 yr (n=2037)	≥60 yr (n=2857)
Lung cancer^a^	117 (1.87)	11 (0.81)	31 (1.52)	75 (2.62)
Stage				
Tis	6 (5.13)	1 (9.09)	0 (0.00)	5 (6.67)
T1a(mi)	34 (29.06)	6 (54.55)	10 (32.26)	18 (24.00)
IA	63 (53.85)	2 (18.18)	19 (61.29)	42 (56.00)
IB	6 (5.13)	2 (18.18)	1 (3.23)	3 (4.00)
IIB	1 (0.85)	0 (0.00)	0 (0.00)	1 (1.33)
IIIA	2 (1.71)	0 (0.00)	1 (3.23)	1 (1.33)
IIIB	1 (0.85)	0 (0.00)	0 (0.00)	1 (1.33)
IV	4 (3.42)	0 (0.00)	0 (0.00)	4 (5.33)
Histological type				
Adenocarcinoma	113 (96.58)	11 (100.00)	29 (93.55)	73 (97.33)
Squamous cell carcinoma	0 (0.00)	0 (0.00)	0 (0.00)	0 (0.00)
Small cell carcinoma	0 (0.00)	0 (0.00)	0 (0.00)	0 (0.00)
Unknown	4 (3.42)	0 (0.00)	2 (6.45)	2 (2.67)

^a^: P value of lung cancer detection rate between 40-49 yr and 50-59 yr is 0.091, 50-59 yr and ≥60 yr is 0.012, 40-49 yr and ≥60 yr is <0.001.

**图 2:**
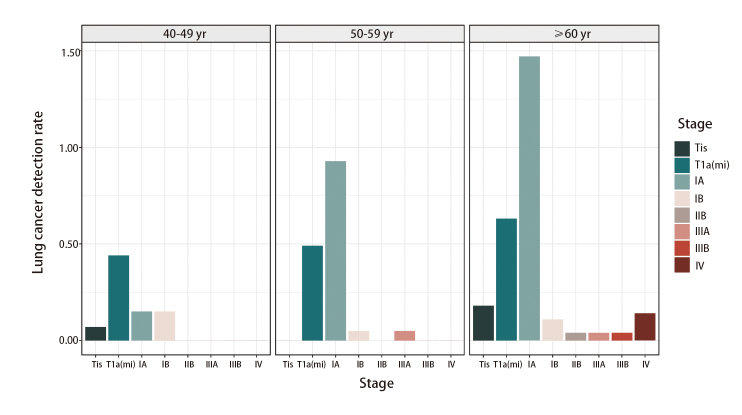
2015-2021年各年龄段女性各期肺癌检出率

### 2.2 单因素Logistic回归分析

表2为筛查人群的详细人口统计学特征。与未确诊肺癌组[（57.44±8.77）岁]相比，确诊组平均年龄更高[（60.82±7.69）岁]（P<0.001）。值得注意的是，确诊组从不吸烟的比例（98.29%）比未确诊组（97.72%）高（P>0.999），而确诊组有二手烟暴露的比例（70.94%）比未确诊组（69.54%）高（P>0.999），但均未发现统计学差异（分类变量各分类P值表中未显示）。以肺癌诊断（不含/含疑似病例）为因变量，调查问卷因素如年龄、恶性肿瘤家族史、个人恶性肿瘤史、疾病史、吸烟情况、饮食习惯、职业暴露、室内环境暴露等109个因素作为自变量进行单因素Logistic回归分析。分析不含疑似病例的结果（表3）显示肺癌的危险因素（P<0.05）有个人恶性肿瘤史（OR=5.24, 95%CI: 3.11-8.82, P<0.001）、年龄≥60岁（OR=3.31, 95%CI: 1.75-6.26, P<0.001）、有毒物质职业暴露[有毒物质定义为砷（吡酸）、石棉、油漆、镍/镉/铬（冶炼或电镀）、橡胶、煤尘、粉尘 、农药、放射线、铍、铀、氡等]（OR=1.55, 95%CI: 1.04-2.29, P=0.035）以及小时候家中做饭以煤为主要燃料（OR=1.93, 95%CI: 1.13-3.28, P=0.014）；而肺癌的保护因素（P<0.05）有因室内外温度变化过敏（定义为由突然的室内外温度变化引起的鼻、咽部症状，如鼻塞、鼻发痒、咽干等^[46]^）（OR=0.45, 95%CI: 0.23-0.86, P=0.016）。表3仅展示部分结果，完整结果见表S1（http://www.lungca.org/files/2024s23s1.pdf），含疑似病例的结果见表S2（http://www.lungca.org/files/2024s23s2.pdf）。

### 2.3 LASSO惩罚Logistic回归分析

LASSO惩罚Logistic回归分析结果显示：年龄（OR=1.07, 95%CI: 1.06-1.07）、个人恶性肿瘤史（OR=3.29, 95%CI: 3.22-3.37）、纺织职业（OR=1.10, 95%CI: 1.08-1.13）、小时候使用煤为燃料（OR=1.14, 95%CI: 1.13-1.16）以及食物过敏史（OR=1.10, 95%CI: 1.07-1.13）是该地区女性肺癌的高危因素。与较低龄女性相比，该地区高龄女性的肺癌发病风险更高；有个人恶性肿瘤史、纺织职业、小时候使用煤为燃料及有食物过敏史与更高的肺癌发病风险有关（见图3和表4）。训练集AUC=0.66（95%CI: 0.61-0.72），验证集AUC=0.62（95%CI: 0.50-0.73），模型如下：肺癌风险=-5.398+0.063*年龄+1.192*个人肿瘤史+0.098*纺织+0.133*小时候家中做饭使用煤为主要燃料+0.092*食物过敏史。

**图 3:**
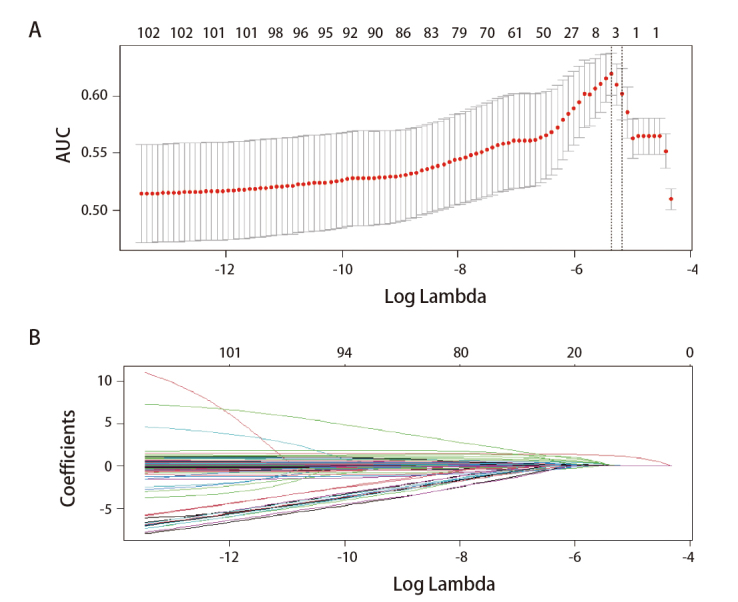
LASSO路径图。根据正则化参数lambda（x轴，Log Lambda）的值，显示出了AUC（A）和所包含变量的系数（B）。虚线标记了最终选择的lambda值0.00469，AUC为0.62。

**表 3:** 单因素Logistic回归分析部分结果（不含疑似病例）

Variables	n	Lung cancer [n (%)]	OR (95%CI)	P
Personal history of cancer				
No	6000	98 (1.63)	Ref	
Yes	225	18 (8.00)	5.24 (3.11-8.82)	<0.001
Missing	31	1 (3.23)	-	-
Family history of cancer				
No	4395	76 (1.73)	Ref	
Other cancer	1071	23 (2.15)	1.25 (0.78-2.00)	0.358
Lung cancer	644	18 (2.80)	1.63 (0.97-2.75)	0.064
Missing	146	0 (0.00)	-	-
Age				
40-49 yr	1362	11 (0.81)	Ref	
50-59 yr	2037	31 (1.52)	1.90 (0.95-3.79)	0.069
≥60 yr	2857	75 (2.63)	3.31 (1.75-6.26)	<0.001
BMI (kg/m^2^)				
<18.5	287	6 (2.09)	Ref	
18.5-23.9	3754	72 (1.92)	0.92 (0.40-2.13)	0.838
24.0-27.9	1793	33 (1.84)	0.88 (0.37-2.12)	0.772
≥28.0	394	6 (1.52)	0.72 (0.23-2.27)	0.580
Missing	28	0 (0.00)	-	-
Education				
No schooling/Primary school	530	12 (2.26)	Ref	
Middle school	3650	70 (1.92)	0.84 (0.45-1.57)	0.592
Specialized school	1017	22 (2.16)	0.95 (0.47-1.94)	0.898
College+	1056	13 (1.23)	0.54 (0.24-1.19)	0.125
Missing	3	0 (0.00)	-	-
Marital status				
Single	192	4 (2.08)	Ref	
Newly married	5507	101 (1.83)	0.88 (0.32-2.41)	0.801
Divorced	238	5 (2.10)	1.01 (0.27-3.81)	0.990
Digamous	69	2 (2.90)	1.40 (0.25-7.84)	0.700
Widowed	248	5 (2.02)	0.97 (0.26-3.65)	0.961
Missing	2	0 (0.00)	-	-
Smoking status				
Yes	142	2 (1.41)	Ref	
No	6114	115 (1.88)	1.34 (0.33-5.49)	>0.999
Smoking frequency				
<30 pack-yr	97	2 (2.06)	Ref	
≥30 pack-yr	20	0 (0.00)	0.98 (0.95-1.01)	>0.999
Missing	6139	115 (1.87)	-	-
Smoking cessation				
No	92	1 (1.09)	Ref	
Yes	36	1 (2.78)	2.60 (0.16-42.72)	0.485
Missing	6128	115 (1.88)	-	-
Variables	n	Lung cancer [n (%)]	OR (95%CI)	P
Years since smoking cessation				
≥15 yr	20	0 (0.00)	Ref	
<15 yr	15	1 (6.67)	1.07 (0.94-1.23)	0.429
Missing	6221	116 (1.86)	-	-
Second-hand smoking status				
No	1784	34 (1.91)	Ref	
Yes	4352	83 (1.91)	1.00 (0.669-1.497)	>0.999
Missing	120	0 (0.00)	-	-
Business (Longest occupation)				
No	6098	112 (1.84)	Ref	
Yes	84	4 (4.86)	2.67 (0.96-7.42)	0.072
Missing	74	1 (1.35)	-	-
Spinning (Longest occupation)				
No	5996	109 (1.82)	Ref	
Yes	186	7 (3.76)	2.11 (0.97-4.60)	0.088
Missing	74	1 (1.35)	-	-
Exposure to toxic substances				
No	4793	80 (1.67)	Ref	
Yes	1447	37 (2.56)	1.55 (1.04-2.29)	0.035
Missing	16	0 (0.00)	-	-
The situation of cooking fumes in the housing during the past 10 years				
No	488	4 (0.82)	Ref	
Little	5260	104 (1.98)	2.44 (0.90-6.65)	0.081
Some	474	9 (1.90)	2.34 (0.72-7.66)	0.159
Quite a lot	34	0 (0.00)	0	0.998
The main fuel used for cooking at home in childhood				
Other^#^	1452	16 (1.10)	Ref	
Coal	4804	101 (2.10)	1.93 (1.13-3.28)	0.014
Allergy to temperature change				
No	5186	107 (2.06)	Ref	
Yes	1070	10 (0.93)	0.45 (0.23-0.86)	0.016
Food allergy				
No	6075	111 (1.83)	Ref	
Yes	181	6 (3.31)	1.84 (0.80-4.25)	0.154
Having a cat or dog at home				
No	5159	104 (2.02)	Ref	
Yes	1095	13 (1.19)	0.58 (0.33-1.04)	0.066
Missing	2	0 (0.00)	-	-
Coronary heart disease				
No	6006	108 (1.80)	Ref	
Yes	250	9 (3.60)	2.04 (1.02-4.08)	0.052*
Cerebral hemorrhage				
No	6251	116 (1.86)	Ref	
Yes	5	1 (20.00)	13.22 (1.47-119.21)	0.090*

P value was derived from the one-way ANOVA analyses between reference categories of each variables and other categories, and missing values were excluded from the analysis; ^#^: including liquefied petroleum gas or coal gas, firewood, electricity, etc; *P value is the result of Fisher’s exact test.

**表 4:** LASSO惩罚Logistic回归分析结果（不含疑似病例）

Variables	Coefficient	OR (95%CI)	Value
Intercept	-5.398		
Age	0.063	1.07 (1.06-1.07)	Age=1 40-49 yr Age=2 50-59 yr Age=3 ≥60 yr
Personal cancer history	1.192	3.29 (3.22-3.37)	1-Yes; 0-No
Spinning	0.098	1.10 (1.08-1.13)	1-Yes; 0-No
Used coal for cooking at home in childhood	0.133	1.14 (1.13-1.16)	1-Yes; 0-No
Food allergy	0.092	1.10 (1.07-1.13)	1-Yes; 0-No

含疑似病例结果见表S3（http://www.lungca.org/files/2024s23s3.pdf），被选择的因素有年龄（OR=1.03, 95%CI: 1.02-1.03）、个人肿瘤史（OR=2.11, 95%CI: 2.05-2.16）以及纺织职业（OR=1.69, 95%CI: 1.64-1.73）。

## 3 讨论

本研究中，在没有烟草等致癌物作为限制的入选标准情况下，该女性群体的肺癌检出率为1.87%（117例），其中大部分检出病例为早期肺癌。此外，我们在该队列中探索肺癌的高危因素，发现年龄、个人恶性肿瘤史、纺织职业、小时候使用煤为燃料和食物过敏史是该地区女性人群肺癌的高危因素，有助于识别该地区的肺癌高危人群。

在本研究的LDCT基线筛查结果中，女性阳性结节检出率为19.63%，高于安徽合肥（4.52%）^[47]^、四川成都（10.00%）^[48]^、河北（11.14%）^[49]^、辽宁（7.72%）^[50]^、重庆（11.36%）^[51]^、云南昆明（6.00%）^[52]^、河南（5.02%）^[53]^等地，也高于全国城市地区平均水平（11.36%）^[54]^。根据本项目纳入与排除标准，我们考虑该差异与人群地理差异、地区经济及医疗机构的诊疗水平^[51,54]^以及对阳性结节定义的不同标准有关。最终117例确诊为肺癌，其中肺腺癌有113例，占比高达96.58%。与上海^[55,56]^、云南个旧^[57]^肺癌早筛项目的病理诊断结果一致，腺癌均远多于鳞癌、小细胞癌和大细胞癌。各年龄段的T1a(mi)期及IA期检出率均最高，合计占肺癌确诊总数的82.91%，此期的高检出率体现了肺癌早筛早诊早治的意义。

我们创新性地采用单因素与LASSO惩罚Logistic回归分析探究女性群体的肺癌高危因素，并建立风险预测模型。结果表明该地区女性肺癌的高危因素包括年龄、个人恶性肿瘤史、纺织职业、小时候家中使用煤为燃料以及食物过敏。然而预测模型训练集AUC仅为0.66，验证集AUC仅为0.62，我们推测由于肺癌的发病较复杂，受遗传、环境等多因素影响，选择的模型可能过于简单而不能充分描述具有复杂结构的数据。其次，训练集的样本量为4379例，较小的样本量降低了模型的泛化能力。同时，在本研究中，只有病理活检确诊的患者才被纳入患癌组，40例高度怀疑肺癌但拒绝进行病理活检的患者被纳入无肺癌组，并且在高度怀疑肺癌而接受病理活检的人群中，7例确诊为转移癌的患者也被纳入无肺癌组，可能进一步降低了模型的AUC。另外，在6256名入组的人群中，仅117例被病理确诊为肺癌，不平衡的类分布可能也导致模型偏向多数类，从而影响AUC的计算结果。尽管有研究^[58,59]^表明由于累积纤维暴露及国际公认致癌物石棉暴露，纺织工人的肺癌风险显著增高，但也有不少队列研究^[60⇓-62]^提示纺织工人的肺癌风险显著降低，结论与本研究相反，可能与纺织粉尘导致的大量的内毒素暴露并引起的先天免疫激活及适应性免疫相关^[63⇓⇓-66]^。因此，纺织职业与肺癌的关联有待进一步探索。烹饪产生的吸入性煤烟和煤烟尘中含有大量多环芳烃，如苯并氟蒽、苯并芘等，因其具有高致突变性而被国际癌症研究机构（International Agency for Research on Cancer, IARC）列为致癌物^[67,68]^。Hosgood、Zhao等^[69,70]^的研究也发现了室内煤烟及煤烟尘暴露可导致肺癌发病风险增高。食物过敏对肺癌发病的影响很少被报道，我们推测它对肺癌的影响与慢性免疫刺激增加癌症风险有关^[71]^，并且可能与过敏反应发生的次数与症状的严重程度有关，具体机制还有待进一步研究。由于小时候家中使用煤为燃料及食物过敏在含疑似病例的模型中被排除，因此应慎重考虑其结果的稳健性。

**表 2:** 筛查人群的人口统计学特征

Characteristic		Non-lung cancer (n=6139)	Lung cancer (n=117)	P
Gender [n (%)]				
Female		6139 (100.00)	117 (100.00)	
Age [n (%)]				<0.001
40-49 yr		1351 (22.01)	11 (9.40)	
50-59 yr		2006 (32.68)	31 (26.50)	
≥60 yr		2782 (45.32)	75 (64.10)	
Personal history of cancer [n (%)]				<0.001
No		5902 (96.14)	98 (83.76)	
Yes		207 (3.37)	18 (15.38)	
Missing		30 (0.49)	1 (0.85)	
Family history of cancer [n (%)]				0.152
No		4319 (70.35)	76 (64.96)	
Other cancer		1048 (17.07)	23 (19.66)	
Lung cancer		626 (10.20)	18 (15.38)	
Missing		146 (2.38)	0 (0.00)	
Smoking status [n (%)]				0.864
Current smoker		91 (1.48)	1 (0.85)	
<30 pack-yr		66 (1.08)	1 (0.85)	0.828
≥30 pack-yr		17 (0.28)	0 (0.00)	
Missing		8 (0.13)	0 (0.00)	
Former smoker		35 (0.57)	1 (0.85)	
Smoking cessation ≥15 yr		20 (0.33)	0 (0.00)	0.487
Smoking cessation <15 yr		14 (0.23)	1 (0.85)	
Missing		1 (0.02)	0 (0.00)	
Never smoker		5999 (97.72)	115 (98.29)	
Missing		14 (0.23)	0 (0.00)	
Second-hand smoking status [n (%)]				>0.999
No		1750 (28.51)	34 (29.06)	
Yes		4269 (69.54)	83 (70.94)	
Missing		120 (1.95)	0 (0.00)	
BMI (kg/m^2^) [n (%)]				0.943
<18.5		281 (4.58)	6 (5.13)	
18.5-23.9		3682 (59.98)	72 (61.54)	
24.0-27.9		1760 (28.67)	33 (28.21)	
≥28.0		388 (6.32)	6 (5.13)	
Missing		28 (0.46)	0 (0.00)	
Education [n (%)]				0.345
No schooling/Primary school		518 (8.44)	12 (10.26)	
Middle school		3580 (58.32)	70 (59.83)	
Specialized school		995 (16.21)	22 (18.80)	
College+		1043 (16.99)	13 (11.11)	
Missing		3 (0.05)	0 (0.00)	
Marital status [n (%)]				0.965
Single		188 (3.06)	4 (3.42)	
Newly married		5406 (88.06)	101 (86.32)	
Divorced		233 (3.80)	5 (4.27)	
Digamous		67 (1.09)	2 (1.71)	
Widowed		243 (3.96)	5 (4.27)	
Missing		2 (0.03)	0 (0.00)	

P value is derived from the univarable association analyses between each of the variables and lung cancer status, missing values were excluded from the comparisons. BMI: body mass index.

本研究中我们未观察到吸烟及二手烟与肺癌发病风险的关联，这与辽宁的大规模肺癌筛查结果^[50]^是一致的。在中国，表皮生长因子受体（epidermal growth factor receptor, EGFR）突变为NSCLC患者最常见的遗传改变，占比高达39.0%，而EGFR突变通常发生在非/轻度吸烟的NSCLC肺癌患者，因此在我国的肺癌筛查中鲜有发现吸烟与肺癌发病显著相关^[72]^。另外，近年来越来越多的证据表明烟草烟雾暴露在我国女性群体中相对少见，而在中老年女性群体中大量生物燃料以及厨房油烟暴露与较高的肺癌发病风险相关^[73,74]^。但在本研究中我们未观察到厨房油烟暴露与较高的肺癌发病风险相关，一方面可能是由于抽油烟机的普及以及居民健康意识的提升使得住房通风条件改善，导致重度油烟暴露样本量较少（34例，0.54%），另一方面可能是油烟暴露对肺癌的发病具有时间滞后效应^[75]^，而本研究中采用的LASSO惩罚Logistic回归模型无法观察到时间的影响。

本研究也有以下局限性。首先，本研究中心单一，将本文研究结果推广到中国其他人群时应谨慎；其次，这是一项单臂队列研究，本研究显示的高危因素还需在多中心中验证；第三，与任何临床研究一样，在本次筛查中不能排除选择偏倚，自愿接受筛查的个体可能在行为和其他影响肺癌发展的因素上存在差异；此外，筛查的样本量和危险因素的暴露人数有限，有可能导致未能发现更多女性肺癌的高危因素。同时，当前国内的肺癌筛查项目还面临着可能造成受检者焦虑、恐慌以及过度医疗的问题，对于检出率达19.63%的阳性结节，根据Fleischner协会肺结节管理建议和Lung-RADs肺结节管理指南^[37,76]^，我们未对所有检出的肺结节行有创病理检查以确定肺癌检出率，这可能低估了LDCT的筛查能力，与此同时可避免过度诊疗^[77]^。另外，我们还将通过后续高危因素的探索完善针对不同人群的肺癌风险预测模型，以期在未来更为精准地识别高危人群。

综上，广州市越秀区采用LDCT筛查女性可检出大量早期肺癌，有助于早诊早治。本研究结果提示年龄、个人恶性肿瘤史、纺织职业、小时候使用煤为燃料和食物过敏史是该地区女性人群肺癌的高危因素，需给予重点关注，以提高该人群对肺癌早筛的重视，并且加强对纺织行业工人职业病的防护。本研究结果为完善肺癌风险预测模型与早诊早治工作提供了科学依据。
